# CD4^+^ T Cell-Mimicking Nanoparticles Broadly Neutralize HIV-1 and Suppress Viral Replication through Autophagy

**DOI:** 10.1128/mBio.00903-20

**Published:** 2020-09-15

**Authors:** Gang Zhang, Grant R. Campbell, Qiangzhe Zhang, Erin Maule, Jonathan Hanna, Weiwei Gao, Liangfang Zhang, Stephen A. Spector

**Affiliations:** a Division of Infectious Diseases, Department of Pediatrics, University of California, San Diego, La Jolla, California, USA; b Department of Nanoengineering, University of California, San Diego, La Jolla, California, USA; c Moores Cancer Center, University of California, San Diego, La Jolla, California, USA; d Rady Children’s Hospital, San Diego, California, USA; Université de Montréal; CIML

**Keywords:** HIV, nanoparticle, autophagy, neutralization, CD4^+^ T cell, macrophage, phospholipase D, human immunodeficiency virus, neutralizing antibodies, phospholipase

## Abstract

Therapeutic strategies that provide effective and broad‐spectrum neutralization against HIV-1 infection are highly desirable. Here, we investigate the potential of nanoengineered CD4^+^ T cell membrane-coated nanoparticles (TNP) to neutralize a broad range of HIV-1 strains. TNP displayed outstanding neutralizing breadth and potency; they neutralized all 125 HIV-1-pseudotyped viruses tested, including global subtypes/recombinant forms, and transmitted/founder viruses, with a geometric mean 80% inhibitory concentration (IC_80_) of 819* *μg ml^−1^ (range, 72 to 8,570 μg ml^−1^). TNP also selectively bound to and induced autophagy in HIV-1-infected CD4^+^ T cells and macrophages, while having no effect on uninfected cells. This TNP-mediated autophagy inhibited viral release and reduced cell-associated HIV-1 in a dose- and phospholipase D1-dependent manner. Genetic or pharmacological inhibition of autophagy ablated this effect. Thus, we can use TNP as therapeutic agents to neutralize cell-free HIV-1 and to target HIV-1 gp120-expressing cells to decrease the HIV-1 reservoir.

## Abstract (precis)

HIV-1 is a major global health challenge. The development of an effective vaccine and/or a therapeutic cure is a top priority. The creation of vaccines that focus an antibody response toward a particular epitope of a protein has shown promise, but the genetic diversity of HIV-1 hinders this progress. Here we developed an approach using nanoengineered CD4^+^ T cell membrane-coated nanoparticles (TNP). Not only do TNP effectively neutralize all strains of HIV-1, but they also selectively bind to infected cells and decrease the release of HIV-1 particles through an autophagy-dependent mechanism with no drug-induced off-target or cytotoxic effects on bystander cells.

## INTRODUCTION

The development of an effective vaccine and/or a therapeutic cure is a top priority for human immunodeficiency virus (HIV) type 1. However, the genetic diversity of this virus, which reaches up to 20% for the envelope polyprotein (Env) sequences (1), stymies this progress. During infection, approximately 20 to 30% of HIV-infected individuals develop type-specific cross-neutralizing antibodies. Of these, about 1% develop broad and very potent neutralizing antibodies (bNAbs) against a wide range of genetically diverse HIV subtypes (2–5). Although current vaccine efforts use structure-guided novel immunogen design to develop vaccines that will induce bNAbs similar in structural recognition, breadth, and potency, none, to date, have evoked the desired *in vivo* response due to high antigenic diversity and the dense N-linked glycan armor, which covers almost the entire envelope protein (Env) (6, 7). An alternative is to use bNAbs in passive immunization, with several studies demonstrating the ability of bNAbs to confer protection from infection, reducing both plasma viremia and the pool of latently infected cells through the recognition of HIV Env on the host cell membrane, potentially facilitating fragment crystallizable (Fc)-mediated clearance (4, 8–10). However, resistant virus isolates appeared either before or after passive bNAb therapy, limiting any putative therapeutic effect (11, 12). Moreover, VRC-PG05, the only donor-derived antibody isolated to date that binds to the highly glycosylated silent face of gp120, failed to neutralize 73% of HIV strains tested and had a relatively high mean IC_50_ of 800 μg ml^−1^, leaving uncertain the potential usefulness of this epitope for vaccine design, therapy, or prevention (13). More recently, tandem trispecific and bispecific broadly neutralizing antibodies, such as BiIA-SG, have shown more promise (5).

The absence of curative treatments or a potential vaccine underscores the need for innovative therapeutic approaches. The development of nanoengineering has given rise to a new avenue of HIV treatment and prevention research. Nanoparticles are being assessed as vehicles for antiviral drugs to improve drug tolerability, circulation half‐life, and efficacy and as carriers for delivery to the central nervous system (14–19). They are also being evaluated for the delivery of small interfering RNAs (siRNAs) to silence gene expression in CD4^+^ T cells, macrophages, and dendritic cells, as well as HIV itself (reviewed in reference 20). Nanoparticle‐based vaccine strategies may also enhance both vaccine safety and anti‐HIV immunogenicity through improved immune targeting and combined presentation of an immunogen and adjuvant (17, 21, 22). Lastly, nanoparticles can also directly interfere with and inhibit viral replication through multivalent presentation of small molecules that block viral assembly processes (17, 23) while also selectively killing latently HIV infected resting memory CD4^+^ T cells (24).

As therapeutic nanoparticles are gaining traction for potential HIV treatment and prevention, cell membrane-coated nanoparticles, made by wrapping plasma membranes of natural cells onto synthetic nanoparticle cores, are emerging as a biomimetic platform to treat various diseases (25–32). This unique biomimicry led us to assess this technology as a potential HIV treatment. Synthetic nanoparticles conjugated with receptor proteins of host cells to target bacteria or viruses for neutralization conventionally require protein identification and labor-intensive synthesis. The fabrication of these T cell membrane-coated nanoparticles (TNP) bypasses these issues by using natural cell membranes as building materials. Specifically, we fused the plasma membranes of uninfected CD4^+^ T cells onto poly(lactic‐co‐glycolic acid) (PLGA) cores, and the resulting TNP mimicked the parent CD4^+^ T cells. We demonstrated previously that these TNP neutralize both R5 and X4 laboratory strains of HIV while also inhibiting gp120-induced apoptosis of bystander uninfected cells (33). In this study, we examined the neutralization breadth and potency of these TNP by using a global panel of HIV isolates. We also investigated the potential application of TNP to inhibit HIV replication and to induce cell death in macrophages and CD4^+^ T cells infected with HIV.

## RESULTS

### TNP broadly neutralize a global panel of Env-pseudotyped HIV.

**FIG 1:**
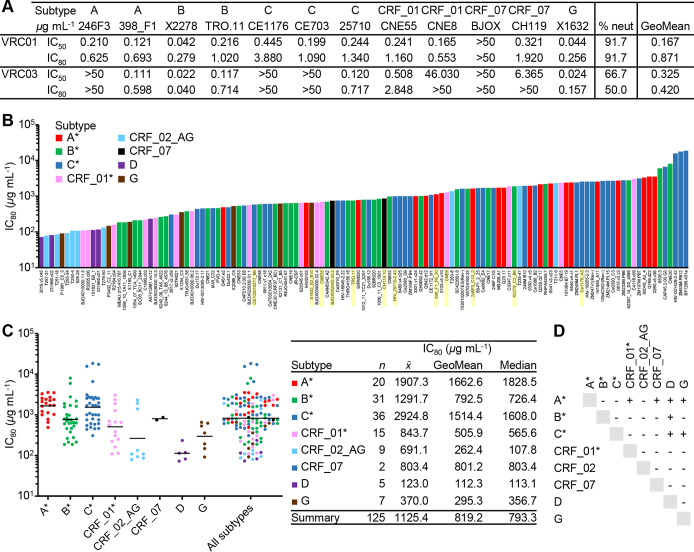
Neutralization by TNP of 125 HIV Env-pseudoviruses across major circulating clades. (A) Neutralization potency (IC_50_/IC_80_) expressed in micrograms per milliliter and neutralization breadths (expressed as percentages) of VRC01 and VRC03 against different HIV Env-pseudoviruses, as tested using the TZM-bl assays. (B) Neutralization potency (IC_80_) expressed as micrograms per milliliter of TNP against different HIV Env-pseudoviruses, as tested using the TZM-bl assays. A* includes subtypes A, A(T/F), AC, and ACD. B* includes subtypes B, B(T/F), and BC. C* includes subtypes C and C(T/F). CRF01* includes CRF01_AE and CRF01_AE(T/F). HIV Env-pseudoviruses shaded yellow were used in the experiment for which results are shown in panel A. (C) (Left) Summary of neutralization potencies (IC_80_) for TNP against the multisubtype pseudoviruses included in panel B. Horizontal bars represent geomean IC_80_ values. (Right) Arithmetic (*x̅*), geometric mean, and median TNP IC_80_ for each subtype. (D) Results of the Games-Howell *post hoc* test after Welch's one-way analysis of variance. For each pair of means, a plus sign indicates a significant difference (*P* < 0.05).

To assess the breadth and potency of TNP to neutralize HIV, we used three standardized panels of viruses: a global multisubtype 109-virus panel that includes transmitted/founder viruses and early/acute infections (34), the global 12-virus panel (35), and the reduced cross-subtype 5-virus panel (36). There was an overlap of viruses among the panels, such that there were 125 unique HIV pseudoviruses tested (Fig. 1). We validated the neutralization protocol using the bNAbs VRC01 and VRC03 against the global 12-virus panel. Against this panel, we observe that the neutralization potencies (geomean 50% inhibitory concentration [IC_50_]/IC_80_) are approximately 0.167/0.871 and 0.325/0.42 μg ml^−1^, respectively, with neutralization breadths of 91 and 50%, respectively, using the IC_50_ in line with previously published observations (37, 38) (Fig. 1A). Conversely, we observed a TNP neutralizing breadth of 100% against the combined 125-virus panel (Fig. 1B). Neutralization potency was robust against all 125 viruses (geometric mean IC_50_/IC_80_, 130.2/819.2* *μg ml^−1^) (Fig. 1C; see also Fig. S1 in the supplemental material). However, subtype preference was evident: there was a statistically significant difference in the TNP neutralization IC_80_ between subtypes as determined by Welch’s one-way analysis of variance (ANOVA) [*F*(7,18) = 23.5; *P* < 0.0001]. The TNP geometric mean IC_80_ values against subtypes B and CRF07 were comparable to the overall geometric mean IC_80_ (792.5 and 801.2* *μg ml^−1^, respectively), whereas the TNP geometric mean IC_80_ was increased 102% against subtype A and reduced by 86% against subtype D (Fig. 1C and D).

10.1128/mBio.00903-20.1 FIG S1 Neutralization of TNP (IC_50_) against 125 HIV Env-pseudoviruses across major circulating clades. (A) IC_50_ (expressed as micrograms per milliliter) of TNP against different HIV Env-pseudoviruses, as tested using the TZM-bl assays. A* includes subtypes A, A(T/F), AC, and ACD. B* includes subtypes B, B(T/F), and BC. C* includes subtypes C and C(T/F). CRF01* includes CRF01_AE and CRF01_AE(T/F). (B) (Left) Summary of IC_50_ for TNP against the multisubtype pseudoviruses included in panel A. The horizontal bar represents the geomean IC_50_ value. (Right) The arithmetic (*x̅*) and geometric mean TNP IC_50_ for each subtype. (C) Results of the Games-Howell *post hoc* test after Welch's one-way analysis of variance. For each pair of means, a plus sign indicates a significant difference (*P* < 0.05). Download FIG S1, TIF file, 0.4 MB. Copyright © 2020 Zhang et al. 2020 Zhang et al. This content is distributed under the terms of the Creative Commons Attribution 4.0 International license.

### TNP targets HIV Env on the surface of the plasma membrane.

**FIG 2:**
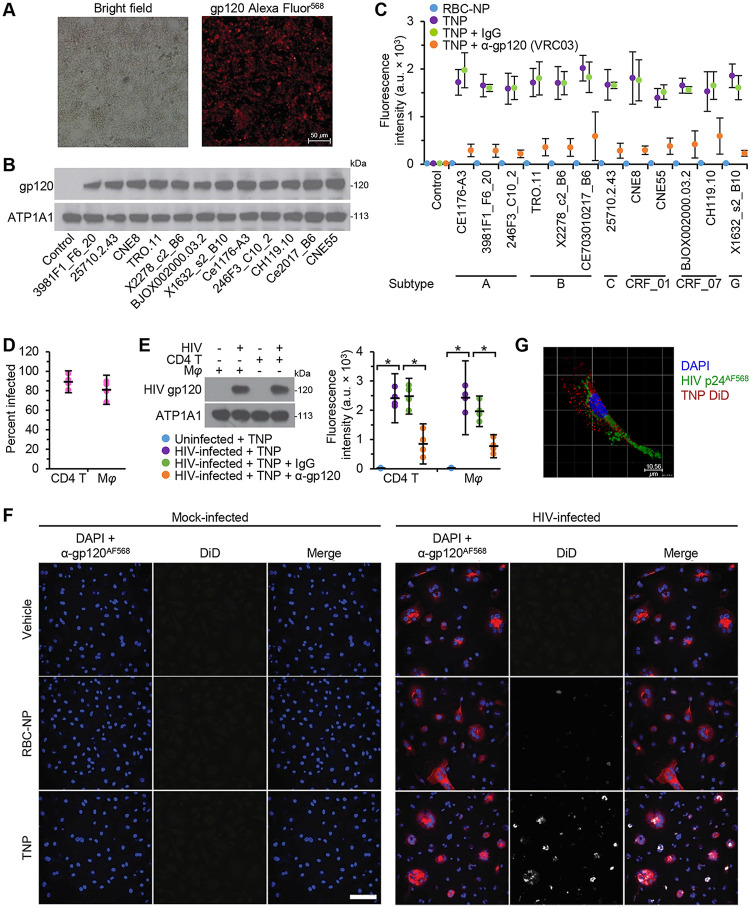
TNP targets cell-associated HIV gp120. (A) HEK 293 T/17 cells were transfected with a panel of Env-expressing plasmids, fixed with 40 mg ml^−1^ paraformaldehyde for 60 min, then probed for the surface expression of HIV gp120, and analyzed by microscopy. Representative images are shown. Bar, 50 μm. (B) The plasma membranes from cells in panel A were harvested and subjected to western blot analysis using antibodies raised against HIV gp120 and ATP1A1 (ATPase Na^+^/K^+^ transporting subunit α1, a loading control). A representative western blot is shown. (C) HEK 293 T/17 cells were transfected with a panel of Env-expressing plasmids, fixed with 40 mg ml^−1^ paraformaldehyde for 60 min, incubated with PBS (vehicle), 1 μg ml^−1^ isotype IgG, or anti-gp120 antibody (VRC03) for 3 h, washed, and then exposed to fluorescently labeled erythrocyte membrane-camouflaged polymeric nanoparticles (RBC-NP) or TNP for an additional 4 h. Cell-attached fluorescently labeled nanoparticles were quantified using a fluorescent plate reader. The experiment was repeated four times, and the mean fluorescence intensity is shown. (D) Primary macrophages (Mφ) and CD4^+^ T cells (CD4 T) were infected with HIV. Cells were fixed, permeabilized, probed for the expression of HIV p24, then visualized and counted using microscopy. (E) Primary macrophages and CD4^+^ T cells were infected with HIV. (Left) The plasma membranes were harvested and subjected to western blotting using antibodies raised against HIV gp120 and ATP1A1. A representative blot is shown. (Right) Uninfected and HIV-infected cells were fixed with 40 mg ml^−1^ paraformaldehyde for 60 min, incubated with PBS (vehicle), 1 μg ml^−1^ isotype IgG, or anti-gp120 antibody (VRC03) for 3 h, washed, and then exposed to fluorescently labeled TNP for an additional 4 h. The cell-attached TNP were quantified using a fluorescent plate reader. *n* = 4. (F) HIV-infected macrophages were exposed to DiD-labeled RBC-NP, DiD-labeled TNP, or PBS (vehicle) for 4 h. Cells were washed, fixed, then probed for HIV gp120, and analyzed using confocal microscopy. Representative images are shown. Bar, 50 μm. *n* = 4. (G) Representative 3D image, obtained by confocal microscopy (z-stack image), of HIV-infected macrophages exposed to TNP for 4 h. Bar, 50 μm. *n* = 4.

We next evaluated the ability of TNP to target HIV-infected cells selectively. As permissively infected cells express HIV gp120 on the cell surface, where it accumulates with newly synthesized viral proteins for HIV packaging, maturation, and budding, we hypothesized that TNP, which possess the natural HIV gp120 receptors (CD4, CCR5, and CXCR4) on their surfaces, will target and bind to cell-associated HIV gp120. Therefore, we transfected HEK 293 T/17 cells with HIV Env plasmids selected from the global pseudovirus panel (Fig. 2A and B) and exposed them to TNP and erythrocyte membrane-camouflaged polymeric nanoparticles (RBC-NP) (Fig. 2C). As expected, the RBC-NP failed to bind either untransfected HEK 293 T/17 cells (control) or HEK 293 T/17 cells expressing HIV gp120. Conversely, the TNP readily bound to HEK 293 T/17 cells expressing HIV gp120, while coculture with the broadly neutralizing antibody VRC03 inhibited this interaction (Fig. 2C). Crucially, an isotype control antibody had no effect on TNP binding to HEK 293 T/17 cells expressing HIV gp120, indicating specificity. Similarly, TNP significantly bound to the surfaces of human primary macrophages and CD4^+^ T cells permissively infected with HIV (Fig. 2D and E). Again, coculture with the broadly neutralizing antibody VRC03 inhibited this interaction (Fig. 2E). Interestingly, TNP preferentially targeted cells expressing HIV gp120 in a heterogeneous population (Fig. 2F), and we observed the internalization of these particles into HIV-infected cells (Fig. 2G).

### Internalized TNP induces autophagy in HIV-infected cells.

**FIG 3:**
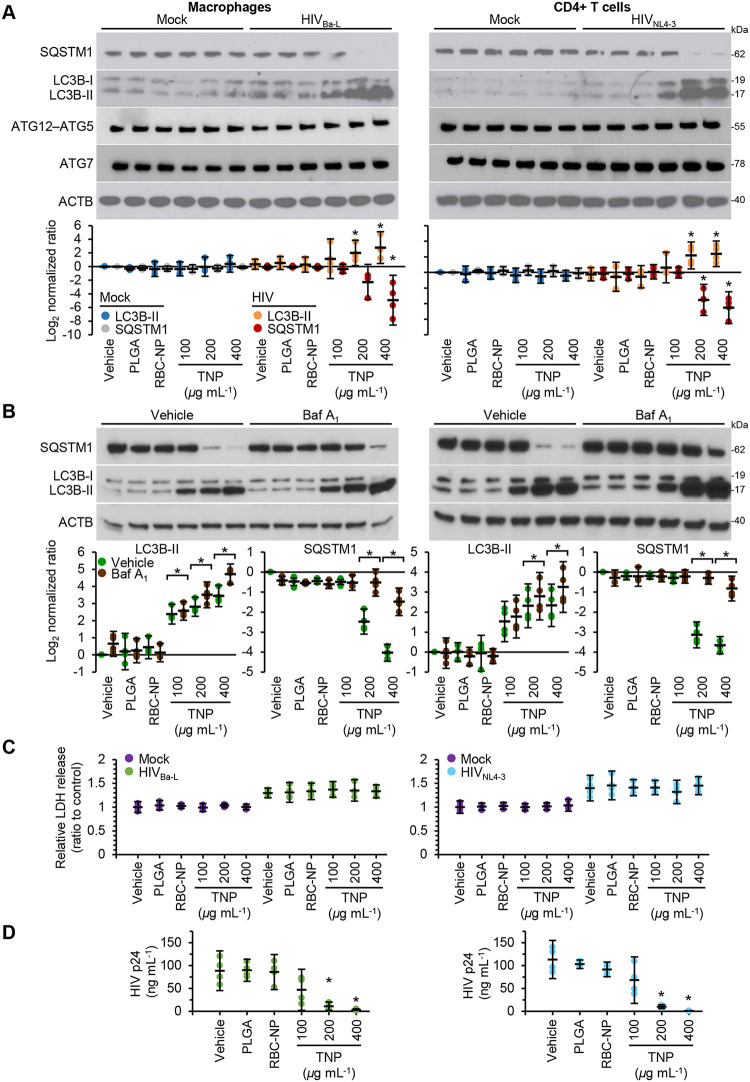
TNP activates autophagy and inhibits HIV replication in macrophages and CD4^+^ T cells. HIV-infected macrophages and CD4^+^ T cells were exposed to 400 μg ml^−1^ PLGA nanoparticles, 400 μg ml^−1^ RBC-NP, or TNP for 4 h, washed three times with PBS, and then incubated for a further 24 h. *n* = 4. (A) (Top) Representative western blots of LC3B isoforms, ATG5, ATG7, and SQSTM1. (Bottom) Densitometric analysis of LC3B and SQSTM1 blots. (B) Cells were treated with vehicle or 100 nM bafilomycin A_1_ for the last 16 h of culture. (Top) Representative western blots of LC3B isoforms and SQSTM1. (Bottom) Densitometric analysis of blots. (C) Aliquots of supernatants were spectrophotometrically tested for LDH as a measure of cell death. (D) An enzyme-linked immunosorbent assay was performed for HIV p24 antigen in the supernatant.

Even in the absence of a drug cargo, internalized PLGA-based nanoparticles can induce macroautophagy (hereafter referred to as autophagy) in immortalized murine bone marrow-derived macrophages and THP1 cells (39). Autophagy is a degradation pathway that occurs at basal levels in all cells and is upregulated in response to stress. Autophagic flux is assessed by monitoring the biogenesis of autophagosomes through a ubiquitin-like system that involves autophagy-related 7 (ATG7) and the ATG12–ATG5 complex, which converts cytosolic microtubule-associated protein 1 light chain 3β-I (MAP1LC3B-I or LC3B-I) to LC3B-II. The ATG12–ATG5 complex then ligates LC3B-II to the nascent autophagosome membrane. The polyubiquitin-binding protein sequestosome 1 (SQSTM1; p62) and SQSTM1-bound polyubiquitinated proteins are incorporated into completed autophagosomes, which then fuse with lysosomes, resulting in the degradation of the engulfed components as well as LC3B-II and SQSTM1 associated with the inner membrane. Thus, the quantification of SQSTM1 and the conversion of LC3B-I to LC3B-II and its turnover are indicators of autophagy maturation (40). Therefore, we analyzed the autophagic effect of TNP on primary leukocytes. Compared with uninfected cells, exposure of infected cells to TNP for 24 h led to a significant dose-dependent increase in LC3B-II and a decrease in SQSTM1 expression, suggesting augmented autophagy (Fig. 3A). To confirm this, we used bafilomycin A_1_, an inhibitor of V-ATPase and thus autophagolysosome fusion and lysosomal degradation (40). LC3B-II and SQSTM1 were both increased, confirming autophagy induction (Fig. 3B). Crucially, neither native nanoparticles (PLGA) nor RBC-NP induced any variation in LC3B-II or SQSTM1 in either uninfected or infected macrophages (Fig. 3A and B). Importantly, since induction of excessive autophagy can lead to cytotoxicity (41), the TNP did not induce significant cell death as measured by lactate dehydrogenase (LDH) release (Fig. 3C) or an increase in visible pyknosis, karyorrhexis, or plasma membrane blebbing.

Since autophagy is necessary for HIV to establish a productive infection (42), and the pharmacological induction of autophagy inhibits HIV release (42–50), we next determined whether TNP influences HIV p24 antigen accumulation in the supernatants of productively infected macrophages and CD4^+^ T cells. TNP induced a dose-dependent decrease in HIV p24 release into the culture supernatants from both macrophages and CD4^+^ T cells in the absence of increased cell death (Fig. 3D) (*P* < 0.05).

### Induction of autophagy by TNP inhibits HIV replication.

**FIG 4:**
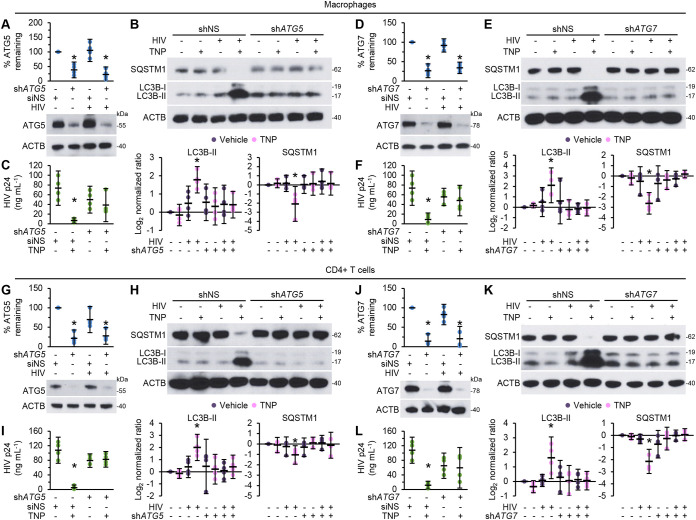
TNP-induced antiviral activity is dependent on autophagy. Mock- and HIV-infected macrophages (A to F) and CD4^+^ T cells (G to L) transduced with *ATG5* shRNA (sh*ATG5*), *ATG7* shRNA (sh*ATG7*), or scrambled shRNA (shNS) were exposed to vehicle or 400 μg ml^−1^ TNP for 4 h, washed three times with PBS, and then incubated for a further 24 h. *n* = 4. (A, D, G, J) (Bottom) Representative western blots of ATG5 or ATG7. (Top) Densitometric analysis of blots. (B, E, H, K) (Top) Representative western blots of LC3B isoforms and SQSTM1. (Bottom) Densitometric analysis of blots. (C, F, I, L) An enzyme-linked immunosorbent assay was performed for HIV p24 antigen in the supernatant.

To determine whether TNP-induced antiviral activity is dependent on autophagy induction, we assessed the effects of TNP on *ATG5*- and *ATG7*-silenced cells (Fig. 4A, D, G, and J). Notably, TNP had no effect on the expression of either ATG5 or ATG7 (Fig. 3A). Both *ATG5* RNA interference (RNAi) and *ATG7* RNAi were effective in silencing their respective genes in both macrophages (Fig. 4A and D) and CD4^+^ T cells (Fig. 4G and J) and were efficient at inhibiting both TNP-induced LC3B lipidation and the degradation of SQSTM1 (Fig. 4B, E, H, and K) and thus autophagy (47). In macrophages, *ATG5* silencing reduced the inhibitory effect of 400 μg ml^−1^ TNP on HIV p24 release from 91% inhibition in scrambled short hairpin RNA (shNS)-transduced cells (*P* = 0.011) to 22.5% inhibition, which was not significantly different from the level for vehicle-treated sh*ATG5* cells (*P* = 0.57) (Fig. 4C), while *ATG7* silencing reduced the inhibitory effect of 400 μg ml^−1^ TNP to just 13.5% (*P* = 0.65) (Fig. 4F). In CD4^+^ T cells, *ATG5* and *ATG7* silencing ablated the inhibitory effect from 92% in shNS cells (*P < *0.005) to an increase of 4.4% in *ATG5*-silenced cells (*P* = 0.73) (Fig. 4I) and a decrease of just 8.5% in *ATG7*-silenced cells (*P* = 0.81) (Fig. 4L).

**FIG 5:**
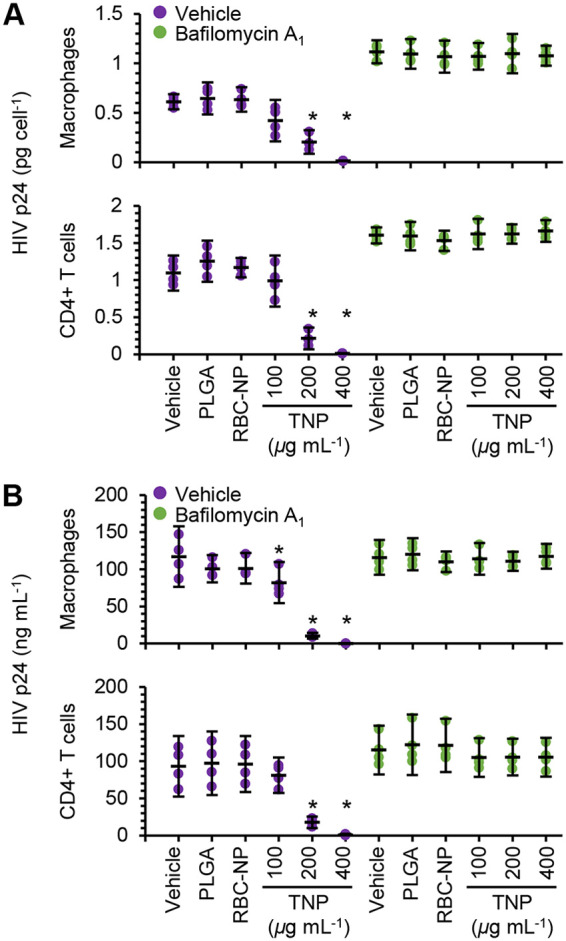
TNP induce the degradation of HIV. HIV-infected macrophages and CD4^+^ T cells were exposed to 400 μg ml^−1^ PLGA nanoparticles, 400 μg ml^−1^ RBC-NP, or TNP for 4 h, washed three times with PBS, and then incubated for 8 h. 100 nM bafilomycin A_1_ was then added, and cells cultured for a further 16 h before they were harvested and lysed. *n *=* *4. Enzyme-linked immunosorbent assays were performed for HIV p24 antigen in the lysate (A) and in the supernatant (B).

We next examined the effect of TNP on intracellular and extracellular HIV p24 antigen in the presence or absence of bafilomycin A_1_. The TNP induced significant decreases in intracellular HIV p24 antigen in both HIV-infected CD4^+^ T cells and macrophages (*P* < 0.05) (Fig. 5A). Importantly, bafilomycin A_1_ ablated the TNP-mediated degradation of HIV, as well as reversing the TNP-mediated inhibition of HIV p24 antigen release (Fig. 5A and B). Collectively, these data suggest that TNP induce the degradation of HIV through the induction of autophagy, which leads to a reduction in the number of released HIV particles.

### PLD1 is an essential regulator for TNP-induced autophagy.

**FIG 6:**
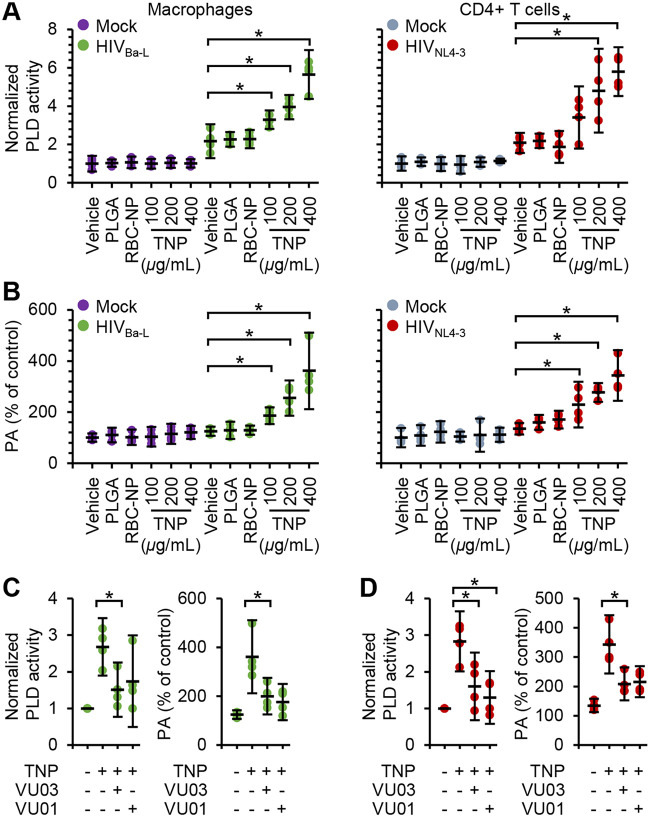
TNP preferentially activate PLD1 in HIV-infected cells. Mock- and HIV-infected macrophages and CD4^+^ T cells were exposed to 400 μg ml^−1^ PLGA nanoparticles, 400 μg ml^−1^ RBC-NP, or TNP for 4 h, washed three times with PBS, and then incubated for a further 24 h before cells were harvested and lysed. *n *=* *4. (A) Lysates were evaluated for PLD activity. (B) Lysates were evaluated for phosphatidic acid (PA) content. (C and D) HIV-infected macrophages (C) and CD4^+^ T cells (D) were pretreated for 1 h with the PLD1 inhibitor VU0359595 or VU0155069 (both at 1 μM) before exposure to 400 μg ml^−1^ TNP. Lysates were evaluated for PLD activity (left) and for PA content (right).

Phospholipase D1 (PLD1) is a major hydrolyzing enzyme located in cytoplasmic and endosomal compartments that can modulate autophagy (51). PLD1 catalyzes the hydrolysis of phosphatidylcholine to choline and phosphatidic acid (PA), a lipid secondary messenger, which binds to the FK506-binding protein (FKBP)-rapamycin-binding (FRB) domain of mechanistic target of rapamycin kinase (MTOR) and is required for mitogen-induced MTOR complex 1 activation. While several enzymes are capable of producing PA, the PA produced by PLD1 is preferentially bound by the FRB domain and thereby activates MTOR activity by displacing the DEP domain containing MTOR interacting protein (DEPTOR), an endogenous inhibitor of MTOR (51). PA also facilitates membrane trafficking, including membrane fusion, since it has a conical shape that packs well in membranes with negative curvature, as would be the case with a site in the process of budding from a donor membrane (52). Therefore, to test the potential function of PLD1 in regulating TNP-mediated autophagy, we monitored the hydrolyzing activity of total PLD in cells with HIV infection and TNP treatment. We observed enhanced PLD activity in both HIV-infected macrophages and HIV-infected CD4^+^ T cells (Fig. 6A) (*P* < 0.033). This translated to a significant increase in PA content in infected cells (Fig. 6B) (*P* < 0.037). We next investigated whether TNP exposure would enhance PLD activity and thus PA content in uninfected and infected cells. TNP had no significant effect on PLD activity in uninfected macrophages or CD4^+^ T cells. Conversely, TNP induced a dose-dependent increase in PLD activity; 400 μg ml^−1^ TNP induced 1.7-fold (standard error of the mean [SEM], ±0.25-fold) (*P* = 0.001) and 1.8-fold (±0.3-fold) (*P* = 0.002) increases in macrophages and CD4^+^ T cells, respectively. This resulted in respective 1.9-fold (±0.3-fold) (*P* = 0.01) and 1.6-fold (±0.3-fold) (*P* = 0.009) increases in PA content in macrophages and CD4^+^ T cells. The TNP-mediated increases in macrophage PLD activity and PA content were reduced to levels approximating those of untreated cells by using two pharmacological inhibitors of PLD, VU0359595 and VU0155069 (Fig. 6C). Surprisingly, although VU0359595 and VU0155069 both decreased the TNP-mediated increase in CD4^+^ T cell PLD activity to levels similar to those in untreated cells (*P* > 0.12), the PA content remained significantly elevated (Fig. 6C) (*P* < 0.043). Importantly, neither PLGA nor RBC-NP induced any significant variation in either PLD activity or PA content.

**FIG 7:**
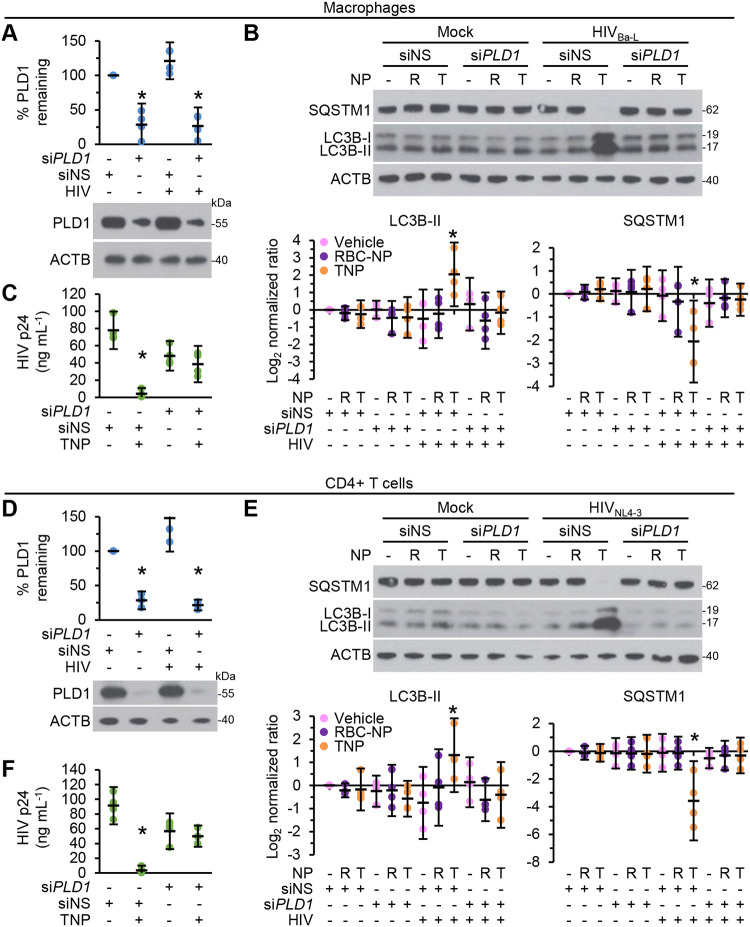
*PLD1* silencing prevents TNP-induced autophagy and decreases in HIV p24 release. Mock- and HIV-infected macrophages (A to C) and CD4^+^ T cells (D to F) transfected with *PLD1* siRNA (si*PLD1*) or scrambled siRNA (siNS) were exposed to vehicle, 400 μg ml^−1^ RBC-NP (R), or 400 μg ml^−1^ TNP (T) for 4 h, washed three times with PBS, and then incubated for a further 24 h. *n* = 4. (A and D) (Bottom) Representative western blots of PLD1. (Top) Densitometric analysis of blots. (B and E) (Top) Representative western blots of LC3B isoforms and SQSTM1. (Bottom) Densitometric analysis of blots. (C and F) Enzyme-linked immunosorbent assays were performed for HIV p24 antigen in the supernatant.

To confirm the role of PLD1 in TNP-induced autophagy and the subsequent decrease in HIV p24 release, we used *PLD1* siRNA (Fig. 7A and D). Blots of cell lysates revealed that *PLD1* silencing inhibited TNP-mediated autophagy, as demonstrated by both LC3B lipidation and SQSTM1 accumulation in both macrophages and CD4^+^ T cells (Fig. 7B and E). *PLD1* silencing also prevented the TNP-mediated reduction of HIV p24 release (*P* > 0.4). These results suggest that PLD1 is important both in the TNP-mediated upregulation of autophagy in HIV-infected cells and in the TNP-mediated decrease in HIV p24 release.

## DISCUSSION

Currently available antiretroviral therapy (ART) has greatly improved life expectancy and quality for those infected with HIV. However, multidrug resistance continues to increase, emphasizing the importance of identifying novel strategies to improve HIV treatment with the goal of reducing the size of the viral reservoir. In the absence of an effective prophylactic vaccine, preventative strategies and novel therapeutics that complement existing options are needed. In the present study, we developed a biomimetic nanoparticle wrapped in a CD4^+^ T cell membrane that not only neutralizes cell-free HIV but also reduces cell-associated HIV through autophagy.

Viral genetic diversity is one of the major obstacles to bNAb-based HIV prevention and immunotherapy, and most bNAbs are ineffective against all circulating strains (2, 5). In contrast, TNP shows excellent binding to HIV gp120 and neutralizes 100% of a global 125-strain pseudovirus panel. Importantly, the application of TNP is independent of prior knowledge of the HIV genetic profile, and the development of TNP does not require the mapping and optimization of antibody epitopes challenged with high levels of somatic hypermutations. Instead, the fabrication of TNP uses biomimetic nanotechnology to target HIV through its host’s natural cognate receptor CD4, and coreceptors CCR5 and/or CXCR4, mimicking the ability of natural CD4^+^ T cells to bind and neutralize HIV. By leveraging the natural affinity of CD4^+^ T cell membrane receptors with HIV, it is possible to overcome the high glycosylation, rapid conformational changes, and steric restriction of the epitopes on HIV envelopes that have limited the conventional development of bNAbs. Moreover. TNP-mediated neutralization is less likely to be affected by genetic mutation, leading to poor efficacy and viral escape. Thus, the use of cell-mimicking nanoparticles to neutralize viral infectivity is a unique approach against HIV, an approach that can potentially become part of cure strategies for many different viral infections, including severe acute respiratory syndrome coronavirus 2 (SARS-CoV-2) (53).

Although the TNP neutralized 100% of our global 125-strain pseudovirus panel, there were statistically significant differences in the TNP neutralization *x̅* IC_80_ between subtypes; in order of the level of neutralization, subtype C was highest, followed by A, B, then D. Interestingly, this matches what is found *in vivo*. Subtype C viruses are slower both in host cell entry by free virus and in cell-to-cell transfer of virus than subtype A and D viruses, and this correlates directly with virulence and more-rapid progression in infected persons: the order of subtypes, from lowest to highest virulence and rapid progression, is subtype C, followed by A, B, and D (54–60). The faster disease progression reported with subtype B and D (which are closely related) infections than with subtype A and C infections may be partially explained by coreceptor tropism; both have a similar propensity to switch to a CXCR4-using phenotype late in disease progression. With substantially better breadth and potency than most bNAbs, TNP are thus a promising candidate for *in vivo* development.

In addition to neutralizing HIV, TNP also inhibited viral release and reduced cell-associated HIV through autophagy. Since we failed to detect autophagy fluctuations post-PLGA or RBC-NP treatment, the induction of autophagy in HIV-infected cells was uniquely attributable to the SUP-T1 cell membranes used to coat the PLGA core of the TNP. Modulation of autophagy processes is an important cellular mechanism for controlling microbial pathogens, and the pharmacologic induction of autophagy potently inhibits HIV (43, 45–48, 50). Illustrating the importance of autophagy in the cellular anti-HIV response, peripheral blood mononuclear cells (PBMC) from elite controllers are more responsive to sirolimus, with treatment leading to an enhanced autophagic response and a greater reduction in virion production (61). However, although autophagy is an innate antiviral defense mechanism, viruses may also hijack autophagy for their efficient replication in a cell type- and virus-specific manner. HIV utilizes autophagosomal membranes as a scaffold for Gag processing and the production of nascent virions (62) while controlling the antiviral proteolytic and degradative late stages of autophagy to avoid its own degradation (62–64). In agreement with this, we did not observe an increase in autophagy in infected cells at our late time point. Nevertheless, we did observe increases in PLD1 activity and PA content, both of which facilitate autophagy (51), membrane trafficking, including membrane fusion and endocytosis (52), and deoxynucleoside triphosphate (dNTP) synthesis through the RAS/MAPK1 and MYC pathways (65), all essential for HIV replication. Importantly, *PLD1* silencing had no effect on LC3B or SQSTM1 accumulation in infected cells, indicating arrested autophagic flux upstream of LC3B lipidation. However, we observed a decrease in released HIV p24 levels, which is consistent with a role for PLD1 in dNTP biosynthesis. Notably, TNP treatment dose-dependently further increased both PLD1 activity and PA content, and this increase inhibited HIV p24 release through a PLD1-dependent degradative autophagy pathway. Targeting host factors used by HIV is a highly attractive antiviral strategy. In contrast to the situation with current antiviral treatments that target viral proteins, the development of resistance to TNP is unlikely, since this strategy minimizes viral escape by working at the host level to degrade both replicating and nonreplicating intracellular HIV through autophagy.

One of the major tasks in HIV cure research is to eliminate infected cells that are disseminated broadly across numerous tissues, including sites that may be relatively inaccessible to host defenses or treatment strategies, such as the lymphatic tissue and the central nervous system, where concentrations of antiviral drugs are lower than in peripheral blood (66, 67). Nanoparticles of 10 to 200 nm can directly enter both the lymphatic tissue and the central nervous system. In this size range, particles of ≤50 nm experience prolonged retention times in the lymph nodes (22), which may enhance both their neutralization activity and inhibition of HIV replication. Therefore, future research will need to resolve empirically the optimal TNP size required for ideal accumulation in lymph nodes and the central nervous system, combined with other properties to balance the TNP pharmacokinetic profile and viral binding efficiency *in vivo* for maximum outcome. Additionally, since previous CD4 and CD4 derivative drug trials failed to yield substantial clinical benefit, in large part because they could not compete with tissue CD4 density for Env binding, future *in vivo* testing will determine whether the TNP will overcome this potential hurdle. Also, for clinical translation, genetic engineering to modify primary human cells, combined with a cell membrane hybridization strategy, will help to mitigate risks of immunogenicity (68).

A common approach to purging the latent HIV reservoir endeavors to reactivate viral production from latently infected reservoir cells, followed by clearance of these cells through a combination of virus- and cell-mediated cytotoxicity, while ART prevents subsequent rounds of infection. However, reactivation of virus may occur only transiently, and reactivation from latency is often insufficient to induce cell death (69, 70). Although they do not induce cell death, TNP encapsulate a biodegradable PLGA nanoparticle at their core—a versatile drug delivery platform that can be loaded with small molecules, peptides, siRNA, or CRISPR-Cas9. Moreover, additional payloads can be loaded into the membrane bilayers, facilitating additional potential mechanisms of viral suppression and/or selective killing of infected cells. Thus, TNP can be used as carriers of cytotoxic cargo that can recognize and kill any cell expressing gp120 on its surface regardless of virus replication competency. In summary, TNP represent a promising drug delivery platform to both neutralize HIV and deliver therapeutic and/or cytotoxic agents specifically to HIV-infected cells while minimizing drug-induced off-target and cytotoxic effects on bystander cells.

## MATERIALS AND METHODS

### HIV.

HIV_NL4-3_ (pNL4-3) was obtained through the NIH AIDS Reagent Program from Malcolm Martin (71). HIV_Ba-L_ was obtained through the NIH AIDS Reagent Program from Suzanne Gartner, Mikulas Popovic, and Robert Gallo (72, 73). Virus stocks were prepared as described previously (74). HIV infectivity was calculated as the 50% tissue culture infectious doses (TCID_50_) as described previously (75), and the multiplicity of infection (MOI) was confirmed using TZM-bl (human; sex: female) (RRID CVCL_B478) cells from John C. Kappes, Xiaoyun Wu, and Tranzyme Inc. (76).

### Cell culture.

Whole blood was drawn from healthy, HIV-seronegative male and female volunteers, aged between 18 and 65 years, at UC San Diego Health Sciences using protocols approved by the Human Research Protections Program of the University of California, San Diego, in accordance with the requirements of the Code of Federal Regulations on the Protection of Human Subjects (45 CFR 46 and 21 CFR 50 and 56). All volunteers gave written informed consent prior to their participation, all samples were deidentified, and donors remained anonymous. PBMC were isolated from whole blood by density gradient centrifugation over Ficoll-Paque Plus (GE Healthcare). Macrophages were prepared and infected with HIV_Ba-L_ at an MOI of 0.1 for 10 days as described previously (63).

CD4^+^ T cells were isolated from PBMC using the CD4^+^ T cell isolation kit (catalog no. 130-096-533; Miltenyi Biotec). Purified resting CD4^+^ T cells were then incubated for 48 h under 5% CO_2_ at 37°C in CD4^+^ T cell medium (RPMI 1640 supplemented with 10% [vol/vol] heat-inactivated fetal bovine serum [FBS] [Sigma], 100 μM nonessential amino acids, 1 mM sodium pyruvate, 0.1 mg ml^−1^ streptomycin, 10^3^ U ml^−1^ penicillin [all from Gibco]) supplemented with 29 nM CCL19 (R&D Systems) and 50 μM 2-sulfanylethan-1-ol (Sigma) before infection with HIV_NL4-3_ at an MOI of 0.1 for 3 h under 5% CO_2_ at 37°C as previously described by the Lewin laboratory (77). CD4^+^ T cells were further cultured for 13 days at 37°C under 5% CO_2_ in CD4^+^ T cell medium supplemented with 5 U ml^−1^ IL-2 (Roche) for a further 13 days prior to experimentation.

HEK 293 T/17 cells (human; sex: female; a kind gift from John Shyy, University of California, San Diego [catalog no. CRL-3216; RRID CVCL_0063; ATCC]) and TZM-bl cells were cultured in Dulbecco’s modified Eagle medium (DMEM) supplemented with 10% (vol/vol) heat-inactivated FBS (Sigma), 0.1 mg ml^−1^ streptomycin, and 100 U ml^−1^ penicillin (all from Gibco) at 37°C under 5% CO_2_. SUP-T1 cells (human; sex: male) (catalog no. CRL-1942; RRID CVCL_1714; ATCC) were cultured in RPMI 1640 supplemented with 10% (vol/vol) heat-inactivated FBS (Sigma), 0.1 mg ml^−1^ streptomycin, and 100 U ml^−1^ penicillin (all from Gibco) at 37°C under 5% CO_2_.

### Formulation of nanoparticles.

PLGA nanoparticle cores were prepared using 0.67 dl g^−1^ carboxy-terminated 50:50 poly(dl-lactide-co-glycolide) (LACTEL Absorbable Polymers) in a solvent displacement process as described previously (25). Human erythrocyte membrane-coated nanoparticles (RBC-NP) were prepared using a three-step process as described previously (29). TNP were fabricated using SUP-T1 cell membranes over a PLGA core by a two‐step process as described previously (33). Fluorescently labeled nanoparticles were fabricated by incorporating 1,1′-dioctadecyl‐3,3,3′,3′‐tetramethylindodicarbocyanine and 4‐chlorobenzenesulfonate salt (DiD; Biotium) with PLGA at 0.1 wt% during the synthesis of the cores. The diameter of the TNP is 105.4 ± 4.4 nm, and the surface zeta potential is −29.5 ± 1.2 mV (33).

### HIV neutralization assay.

The neutralization activities of TNP and bNAbs were assessed using pseudovirus and a single round of replication in TZM-bl cells. Anti-HIV gp120 monoclonal antibodies VRC01 (catalog no. 12033) and VRC03 (catalog no. 12033) were obtained through the NIH AIDS Reagent Program, Division of AIDS, NIAID, NIH, from John Mascola (37). The pseudovirus panel of 125 geographically and genetically diverse Env-pseudoviruses representing the major subtypes and circulating recombinant forms were a kind gift from Dennis R. Burton (Scripps Research Institute, San Diego, CA). This panel includes previously described reference panels of viruses with some overlap between panels, such that there were 125 unique HIV pseudoviruses (34–36). Pseudoviruses were generated by cotransfection of HEK 293 T/17 cells with an Env-expressing plasmid and an Env-deficient genomic backbone plasmid (pSG3ΔEnv) using polyethylenimine (Sigma). Pseudoviruses were collected 48 h posttransfection and were stored at –80°C. TCID_50_ was determined in TZM-bl using Spearman-Karber analysis. Virus neutralization was measured using a luciferase-based assay in TZM-bl cells as described previously (78). Briefly, serial dilutions of TNP or bNAbs (VRC01 or VRC03) were incubated with 200 TCID_50_ of virus in the presence of DEAE-dextran, and neutralizing activity was assessed by measuring luciferase activity after 48 h using a FilterMax F5 multimode microplate reader (Molecular Devices). Dose-response curves were fitted using nonlinear regression to determine IC_50_ and IC_80_ values (Prism, v. 8; GraphPad).

### Cytototoxity and cell viability.

The lactate dehydrogenase (LDH) activity of supernatants was measured using a mixture of diaphorase/NAD^+^ and 3-(4-iodophenyl)-2-(4-nitrophenyl)-5-phenyl-2*H*-tetrazol-3-ium chloride/sodium 2-hydroxypropanoate, and the percentage of cytotoxicity was calculated according to the manufacturer’s protocol (TaKaRa Bio). Cell viability was determined using 3-(4,5-dimethylthiazol-2-yl)-2,5-diphenyltetrazolium bromide according to the manufacturer’s protocol (Sigma).

### Microscopy.

Cells were fixed in Dulbecco’s phosphate-buffered saline (PBS) supplemented with 40 mg ml^−1^ paraformaldehyde for 60 min, and for intracellular staining, they were permeabilized with 0.1% (vol/vol) Triton X-100 for 10 min. Cells were then probed with anti-HIV p24 (catalog no. M0857; RRID AB_2335686; Agilent) or anti-HIV gp120 (catalog no. 20-HG81; RRID AB_231637; Fitzgerald Industries) for 60 min, washed, probed with an Alexa Fluor 568-conjugated donkey anti-goat (catalog no. A11057; RRID AB_2534104; Thermo Scientific) or donkey anti-mouse (catalog no. A10037; RRID AB_2534013; Thermo Scientific) secondary antibody for 30 min, washed, and then counterstained with 4′,6-diamidino-2-phenylindole (DAPI) (Molecular Probes). Labeled cells were visualized using an Olympus FluoView FV-1000 confocal imaging system on an IX81 platform equipped with U Plan Fluorite 10×/0.4-numerical-aperture (NA) and 60×/1.42-NA objectives (Olympus). To quantify infection, 30 random fields were counted for each condition.

### Western blotting.

The following antibodies were used: anti-ACTB (catalog no. A2228; RRID AB_476697; Sigma), anti-ATG5 (catalog no. 2630; RRID AB_2062340; Cell Signaling Technology), anti-ATG7 (catalog no. 2631; RRID AB_2227783; Cell Signaling Technology), anti-ATP1A1 (catalog no. A01483; RRID AB_1968790; GenScript), anti-HIV gp120 (catalog no. 20-HG81; RRID AB_231637; Fitzgerald Industries International), anti-MAP1LC3B (catalog no. NB100-2220; Novus Biologicals), anti-PLD1 (from Cell Signaling Technology [catalog no. 3832; RRID AB_2172256] or Santa Cruz Biotechnology [catalog no. sc-28314; RRID AB_677324]), and anti-SQSTM1 (catalog no. ab56416; RRID AB_945626; Abcam).

Whole-cell lysates were prepared using 20 mM HEPES (Gibco), 150 mM NaCl (Fisher), 1 mM EDTA (Sigma) supplemented with 1% (vol/vol) Triton X-100 (Sigma), and 1% (vol/vol) Halt protease and phosphatase inhibitor cocktail (Thermo Scientific). Membrane fractions were prepared using the Mem-PER plus membrane protein extraction kit (Thermo Scientific). Lysates were resolved using a 2-[bis(2-hydroxyethyl)amino]-2-(hydroxymethyl)propane-1,3-diol-buffered 12% polyacrylamide gel (GenScript) and were transferred to 0.2-μm polyvinylidene difluoride or 0.45-μm nitrocellulose membranes (Thermo Scientific), followed by detection with primary antibodies, horseradish peroxidase-tagged secondary antibodies (Santa Cruz Biotechnology), and the SuperSignal West Dura extended-duration substrate (Thermo Scientific) using CL-XPosure film (Thermo Scientific). Films were then scanned at 16 bit, and the relative densities of the target bands were calculated using ImageJ (NIH) and were compared to the applicable reference band: ACTB (for whole-cell lysates) or ATP1A1 (for membrane fractions). Only bands with transparencies of 2 to 11 (58368-5991 grayscale values) on the Stouffer 21-step transparent guide were used and analyzed.

### TNP-cell-associated gp120 binding assay.

To test the binding of TNP to HIV gp120-expressing HEK 293 T/17 cells or HIV-infected primary cells, cells were fixed using PBS supplemented with 40 g liter^−1^ paraformaldehyde for 60 min and were then incubated with PBS (vehicle), 1 μg ml^−1^ IgG isotype control, or 1 μg ml^−1^ anti-gp120 (VRC03) for 3 h. After a wash with PBS, cells were incubated with 400 μg ml^−1^ DiD-TNP for 4 h. Cells were then extensively washed to remove extraneous cell-free TNP before cell-attached TNP were quantified using a FilterMax F5 multimode microplate reader (Molecular Devices).

### RNAi.

Lentiviral transduction with MISSION lentiviral particles containing short hairpin RNA (shRNA) targeting *ATG5* (catalog no. SHCLNV-NM_004849; clone no. TRCN0000151963) or *ATG7* (catalog no. SHCLNV-NM_006395; clone no. TRCN0000007584), or a scrambled no-target negative control (catalog no. SHC002V) (all from Sigma), was performed as described previously (48, 74). Cells were transfected with *PLD1* siRNA (catalog no. sc-44000; Santa Cruz Biotechnology) or control scrambled siRNA (siNS; catalog no. 4390846; Thermo Scientific) using Lipofectamine RNAiMAX transfection reagent (Thermo Scientific) as described previously (79). Transfection efficiency was assessed with BLOCK-iT Alexa Fluor red fluorescent control (Thermo Scientific) by flow cytometry (79).

### PLD activity and PA production.

PLD1 activity was estimated using the Amplex Red phospholipase D assay kit (Thermo Fisher) according to the manufacturer’s protocol using a FilterMax F5 multimode microplate reader (Molecular Devices). Phosphatidic acid production was determined using the Total Phosphatidic Acid assay kit (Cell Biolabs) according to the manufacturer’s protocol using a FilterMax F5 multimode microplate reader. VU0359595 and VU0155069 were purchased from Santa Cruz Biotechnology and were used at 1 μM for 1 h before treatment.

### Statistics.

Samples were assigned to experimental groups through simple random sampling. Sample size was determined using a 2-sample 2-sided equality test with a power (1 – *β*) of 0.8, an *α* value of 0.05, and preliminary data where the minimum difference in outcome was at least 70%. Sample sizes are given in the figure legends and refer to the number of donors (independent biological replicates [*n*]). Data are represented as dot blots with arithmetic means ± 95% confidence intervals for independent biological replicates and as the arithmetic means ± standard deviations for technical replicates (cell line data). Data were assessed for symmetry, or skewness, using Pearson’s skewness coefficient. Normalized ratiometric data were log_2_ transformed. Comparisons between groups were performed using the paired, two-tailed Student *t* test. *P* values were determined on the basis of biological replicates (with technical replicates averaged within each biological replicate). In all experiments, differences were considered significant when *P* was less than 0.05 (*, *P* < 0.05).
