# Supplementary figures and images for: CD4+ T Cell-Mimicking Nanoparticles Broadly Neutralize HIV-1 and Suppress Viral Replication through Autophagy
Source: mBio. 2020 Sep 15;11(5):e00903-20. doi: 10.1128/mBio.00903-20 (PMC7492730; doi:10.1128/mBio.00903-20)

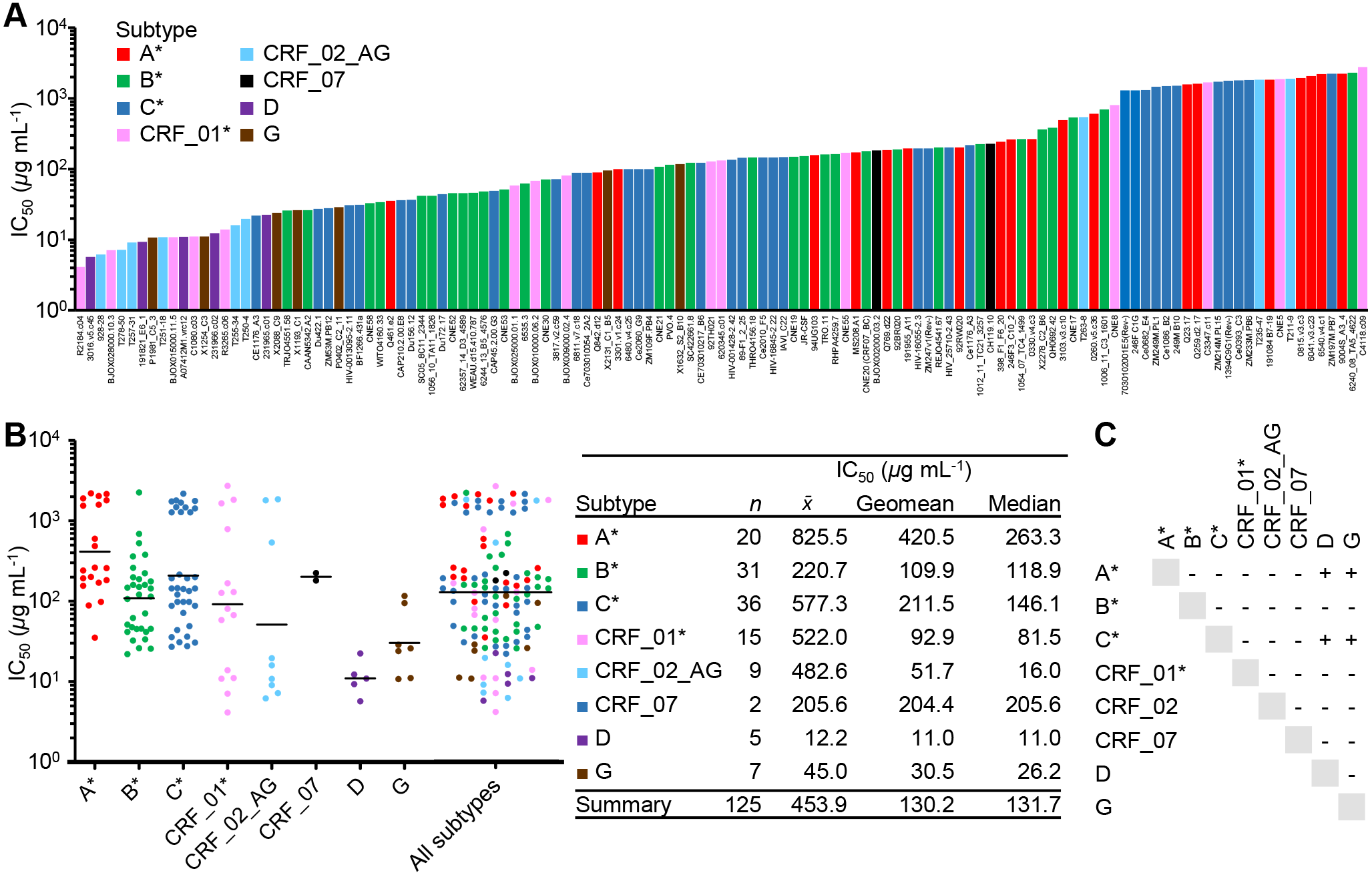

Supplement: FIG S1 [file mBio.00903-20-sf001.tif]
